# Dysregulated *miR*-*645* affects the proliferation and invasion of head and neck cancer cell

**DOI:** 10.1186/s12935-015-0238-5

**Published:** 2015-09-17

**Authors:** Qiang Sun, Shuai Chen, Xue Zhao, Ming Yan, Zheng Fang, Haibin Wang, Junfang Zhao, Minglei Sun, Xinguang Han, Wantao Chen, Xinming Li

**Affiliations:** Department of Stomatology, The First Affiliated Hospital of Zhengzhou University, No. 1, East Jian she Road, Zhengzhou, 450052 Henan China; Institute of Clinical Medicine, The First Affiliated Hospital of Zhengzhou University, No. 1, East Jian she Road, Zhengzhou, 450052 Henan China; Department of Neuroimmunology Research, The Henan Academy of Medical and Pharmacologic Sciences, Zheng-Zhou University, Daxue Rd No. 40, Zhengzhou, 450052 Henan China; Department of Oral and Maxillofacial-Head and Neck Oncology, Ninth People’s Hospital, Shanghai Jiao Tong University School of Medicine, Shanghai, 200011 China

**Keywords:** *miR*-*645*, HNSCC, Growth, Metastasis, Invasion

## Abstract

**Background and purpose:**

Dysregulated miRNAs play an important role in many malignant tumors. However, elucidating the roles of miRNAs in cancer biology, especially in epithelial cancers, remains an ongoing process. In this study, we identified the differentially expressed *miR*-*645* in the progressing of head and neck squamous cell carcinoma (HNSCC) and investigated its biological function.

**Methods:**

The association between clinicopathological parameters and the expression levels of the candidated miRNAs were analyzed by using the Kaplan–Meier survival analysis. The cell growth, invasion and migration potential, and clone formation were observed to detect the functions of the miRNAs in HNSCC cells.

**Results:**

In the 34 HNSCC tissues with lymph node metastasis, the expression level of *miR*-*645* was 0.54 ± 0.12, and the expression level was 0.22 ± 0.05 in the 28 tissues with non lymph node metastasis (*p* = 0.017). In patients with HNSCC, higher level of *miR*-*645* expression significantly correlates with worse overall survival (*p* = 0.04). Ectopic expression of *miR*-*645* promoted cell invasion and migration.

**Conclusions:**

*miR*-*645* play a key role in cell invasion and metastasis and their expression correlates with overall survival in the patients with HNSCC.

## Background

MicroRNAs (miRNAs) are endogenous RNAs that play important gene-regulatory roles in animals via sequence-specific interactions with the 3′UTR of cognate mRNA targets, causing suppression of translation and mRNA decay [1, 2]. Nucleotides 2–7, from the 5′ end of the miRNAs, are referred to as the “seed” and are critical for hybridization to the targets [3], It has been firmly established that miRNAs regulate many key cellular processes such as cell growth, differentiation and apoptosis [4, 5]. Subsets of miRNAs have been identified as potential diagnostic and prognostic markers in malignant tumors [6–8]. Many evidences suggest that the regulatory capacity of miRNAs is dysregulated and exploited in malignant tumors [9]. Several miRNAs are up-regulated in specific tumors appears a general trait of human cancers which playing a causal role in the transformed phenotype [10–12], and the mechanisms are remain to be known further. Although the number of verified human miRNAs is still expanding, the functions of only a few of them have been described.

Head and neck squamous cell carcinoma (HNSCC) ranks sixth among cancers worldwide [13], includes tumors of the oral cavity, oropharynx, and larynx. Survival rates for HNSCC have remained unchanged throughout the last three decades, and half of all cases die within 5 years of diagnosis [11]. The presence of lymph node metastasis affects more than 50 % of HNSCC patients and it is one of the most important prognostic indicators associated with poor long survival rates [14, 15]. There are some biomarkers for the classification diagnosis, individual treatment and prognosis of HNSCC investigated by miRNAs array and studies have shown altered miRNAs profiles in HNSCC compared to their normal tissue [16–21]. *miR*-*645* has been reported to be disrupt-expression in pathological states [22–25], however, the role of *miR*-*645* in metastasis of cancer has not been reported.

In this study, we examined the expression of *miR*-*645* in HNSCC samples by gene chips and further confirmed in HNSCC samples and HNSCC cell lines using real-time PCR. We found that *miR*-*645* levels were up-regulated in HNSCC tissues and highly invasive cell lines. Furthermore, we have investigated the mechanism of *miR*-*645* in HNSCC cancer cell lines. These results show that exogenous overexpression of *miR*-*645* promotes the invasion and migration of HNSCC cells in vitro.

## Methods

### Cell culture

The human HNSCC cell lines, HN4 and HN12 were kindly provided by Shanghai Key Laboratory of Stomatology [26–30]. These cell lines were cultured in DMEM supplemented with 10 % heat-inactivated FBS (GIBCO BRL, NY, USA), penicillin (100 units/ml) and streptomycin (100 μg/ml) at 37 °C in a humidified 5 % CO_2_ atmosphere.

### Tissue samples and reagents

Tissue samples from patients undergoing curative treatment for definitely diagnosed HNSCC were obtained by surgery, with half of each sample quickly frozen in liquid nitrogen and stored at −80 °C until use and the other half embedded in paraffin for pathological examination. All patients selected in the study were informed consent in advance. In parallel, a separate cohort of 47 patients also was assembled from a large pool of patients in the database based on histologic diagnosis of HNSCC who had undergone radical surgery. We retrospectively reviewed the medical records of patients with HNSCC. In this study, we retrospectively reviewed the medical records of patients. Total RNAs were extracted from paraffin blocks using the high pure miRNA isolation kit according to the manufacturer’s protocol (Roche, Switzerland) before further analysis.

Both the *miR*-*645* inhibitor and its mimics were purchased from GenePharma (Shanghai, China). The high pure miRNA isolation kit was purchased from Roche (Basel, Switzerland). The miRcute miRNA qPCR detection kit and miRcute miRNA qPCR detection kit were purchased from TIANGEN BIOTECH (Beijing, China).

### Invasion assays and wound-healing experiment

In vitro invasion assays were performed to analyze the invasive potential. A total of 8 × 10^4^ various cells in 200 μl serum-free DMEM medium were plated onto BD BioCoat™ Matrigel™ Invasion Chamber (8 μm pore size; BD Biosciences) and the lower chamber was immediately filled with 500 μl of DMEM medium with 10 % FBS as a chemoattractant. After 24 h of incubation in a humidified atmosphere containing 5 % CO_2_ at 37 °C, the non-invading cells are removed from the upper surface of the membrane by a cotton swab and the membranes were then fixed with methanol and stained by 0.2 % crystal violet. For wound-healing experiments, cells were plated in 6-well plates, transfected as indicated, and cultured to confluency. Cells were serum-starved and scraped with a P200 tip (time 0), and the number of migrating cells is counted from pictures (5 fields) taken at the indicated time points.

### Clony formation

Twenty-four hours after transfection, HNSCC cells (1 × 10^5^ cells per plate) were plated in 100-mm culture dishes and incubated with 600 μg/ml G418 in final concentration for 14 days to allow colonies formation. The colonies were then washed twice with PBS, fixed with 70 % ethanol and stained with Coomassie Blue. Colonies of more than 50 cells were counted under a dissecting microscope. The data from colony formation were showed as mean ± SD from at least three independent experiments, each being performed in triplicate.

### Statistical analysis

Statistical analyses for real-time PCR and the in vitro analysis were performed with software from SPSS 13.0 (standard version 13.0; SPSS Inc., Chicago, IL, USA). The results of the cell proliferation assay, colony formation assay, and in vitro invasion assay were evaluated by Student’s *t* tests. Patients were divided in two groups based on the median of the *miR*-*645* expression values. Tumors were then classified as high miR-645 group if the expression value was equal to or above the median and as low *miR*-*645* group if the expression value was below the median. The correlation between *miR*-*645* expression and the disease-free survival probability were estimated by using Kaplan–Meier survival analysis. A *P* < 0.05 was taken as the level of significance.

## Results

### The association of *miR*-*645* expression with metastatic rates in patients with HNSCC

**Fig. 1:**
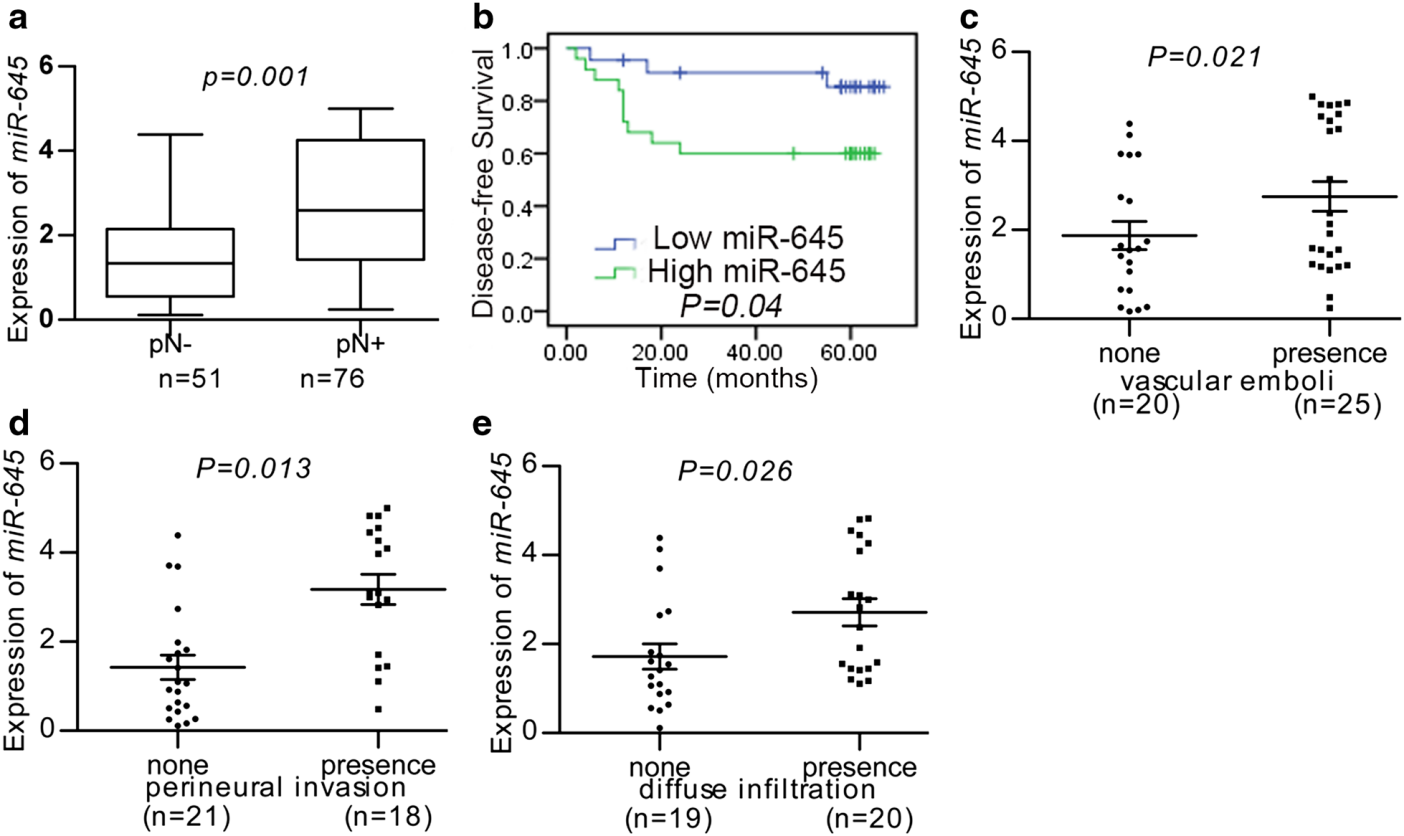
Clinical association of *miR*-*645* with HNSCC patients. **a** Mature *miR*-*645* levels were measured in HNSCC samples by real-time PCR. **b** Kaplan–Meier graph representing the probability of disease-free survival in HNSCC patients from the “Milan-INT” dataset stratified. The log-rank test *P* value reflects the significance of the association between low *miR*-*645* level and disease-free survival. **c**–**e** Mature *miR*-*645* levels were compared between HNSCC samples with severe histological signs (vascular emboli, perineural invasion, diffuse infiltration) and the tissues with non-severe histological signs by real-time PCR

We first measured mature *miR*-*645* levels in a group of tissue specimens from the HNSCC patients. In the 76 HNSCC tissues with lymph node metastasis, the expression level of *miR*-*645* was 2.71 ± 0.24, and the expression level was 1.58 ± 0.23 in the 51 tissues with non lymph node metastasis (*p* = 0.001, Fig. 1a). The results showed that the *miR*-*645* expression level in the primary HNSCC samples with lymph node metastasis was significantly higher than that in the tissues without lymph node metastasis. The correlations between the *miR*-*645* expression level and clinical pathological characteristics of are summarized in Table 1. Statistically significant associations between the *miR*-*645* expression levels and metastatic rates were observed. In the HNSCC tissues with severe histological signs (vascular emboli, perineural invasion, diffuse infiltration), the expression level of miR-645 was also significantly higher than the expression level in the tissues with non-severe histological signs (Fig. 1c–e). However, there was no significant correlation between the expression level of *miR*-*645* and age, sex, tumor size, site, smoking history, alcohol history (Table 1).

**Table 1:** Associations between *miR*-*645* and clinical parameters (n = 62)

Characteristic	No. of patients		miR-645 ∆Ct^a^	*P*
	No.	%	Mean ± SD	
Age, years				
≥60	26	41.9	0.29 ± 0.08	0.173
<60	36	58.1	0.48 ± 0.11	
Sex				
Male	43	69.4	0.37 ± 0.09	0.625
Female	19	30.6	0.45 ± 0.13	
Pathologe grade				
I	26	42.6	0.22 ± 0.076	0.025
II	27	44.3	0.46 ± 0.12	
III	8	13.1	0.82 ± 0.21	
T stage				
T1, 2	27	43.5	0.34 ± 0.08	0.406
T3, 4	34	54.8	0.46 ± 0.11	
N stage				
pN−	28	45.2	0.22 ± 0.05	0.017
pN+	34	54.8	0.54 ± 0.12	
Site				
Tongue	25	41.7	0.32 ± 0.11	0.514
Gingival	12	20.0	0.44 ± 0.18	
Cheek	7	11.7	0.35 ± 0.16	
Floor of mouth	11	18.3	0.34 ± 0.15	
Oropharynx	5	8.3	0.80 ± 0.41	
Histologic signs of severity (vascular emboli, perineural invasion, diffuse infiltration)				
None	26	41.9	0.28 ± 0.05	0.024
Presence	21	33.9	0.60 ± 0.13	
Smoking history				
Nonsmoker	28	45.2	0.41 ± 0.09	0.452
Smoker	30	48.4	0.32 ± 0.07	
Alcohol history				
Nondrinker	38	61.3	0.41 ± 0.08	0.339
Drinker	20	32.3	0.29 ± 0.07	

*SD* standard deviation, *T* tumor stage, *N* lymphnode stage

^a^∆Ct indicates the difference in the cycle number at which a sample’s fluorescent signal passes a given threshold above baseline (Ct) derived from a specific gene compared with that of *U6* in tumor tissues

We next measured mature *miR*-*645* levels in a collection of HNSCC patients with clinical history. Patients were divided in two groups, with, respectively high or low levels of *miR*-*645*. Remarkably, when tested using the Kaplan–Meier survival analysis, the *miR*-*645* “low” group displayed a significant longer disease-free survival when compared to the “high” group (Fig. 1b). These data suggested a possible link between *miR*-*645* expression and tumor progression.

### miR-645 promotes cell proliferation

**Fig. 2:**
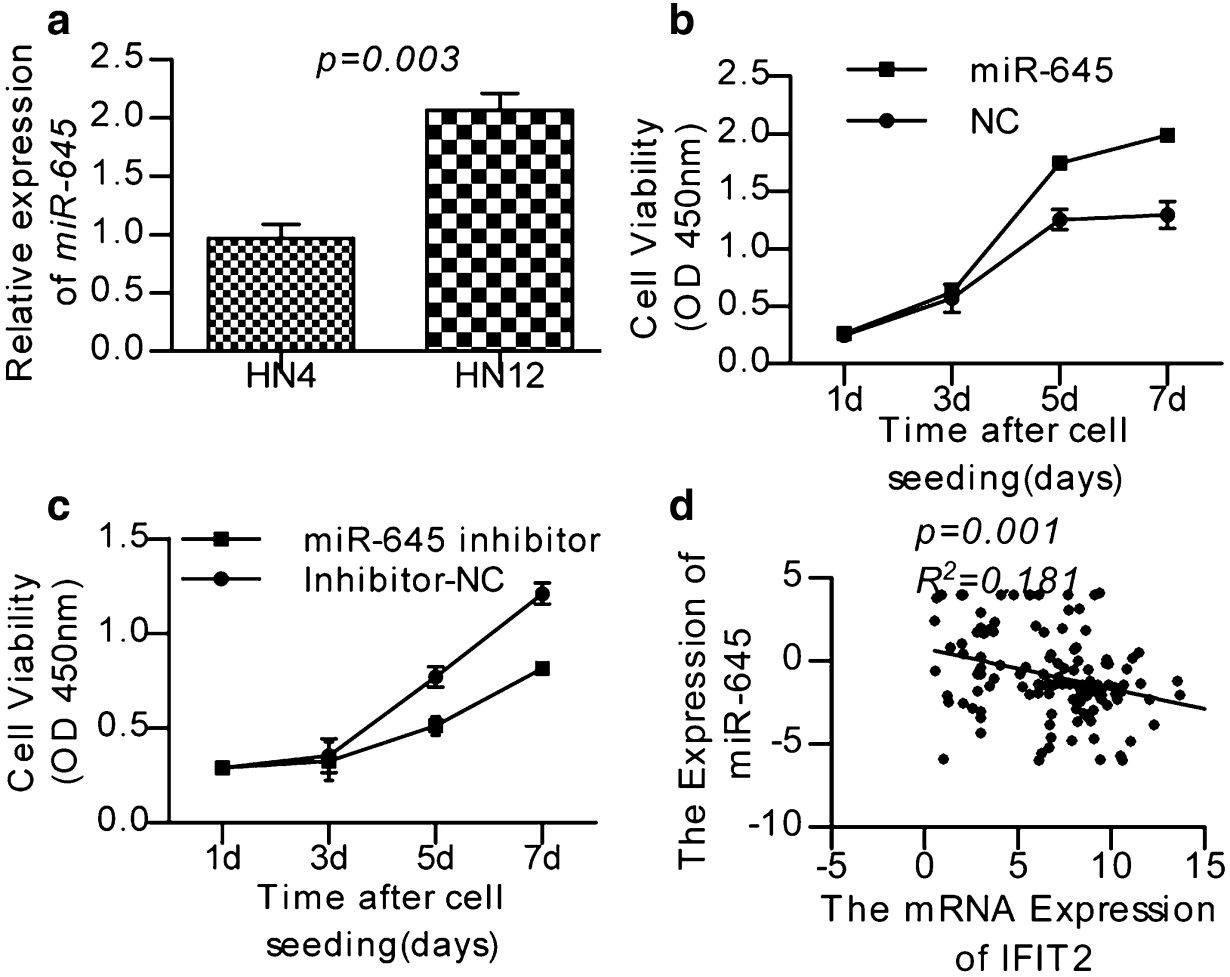
*miR*-*645* promotes HNSCC. **a** Expression levels of *miR*-*645* in cellular models of metastatic progression were tested by real-time PCR. Values related to the nonmetastatic, less aggressive cell line (HN4) are normalised to U6 and shown as the mean and SD. **b**, **c** Cell growth curves: proliferation of phenotypically stable indicated cell lines was monitored by the CCK-8 assay. **d** Linear regression analyse the correlation between miR-645 and IFIT2

HN4 and HN12 cell lines were established from primary HNSCC tissue and the lymph node metastatic tissue from the same patient, respectively. We used these cell lines to investigate how gain or loss of function of *miR*-*645* impacted cell biological behavior. The HN12 cells displayed high migration capacities and contained a relatively high level of *miR*-*645* (Fig. 2a). First, we assessed the growth of *miR*-*645*-transfected and miR-NC-transfected HN4 cells after transient transfection. As shown in Fig. 2b, miR-645 was able to increase the proliferation of *miR*-*645*-transfected cells compared with miR-NC-transfected cells significantly at day 3 and 5 (*P* < *0.05*, Student’s *t* test) (Fig. 2b). We further tested if endogenous expression of *miR*-*645* was required for HNSCC invasion in the cancer cell line HN12. For this purpose, we silenced *miR*-*645* and this treatment led to an approximately 1.5-fold decline in growth properties (Fig. 2c).

### Ectopic expression of *miR*-*645* promotes cancer metastasis

**Fig. 3:**
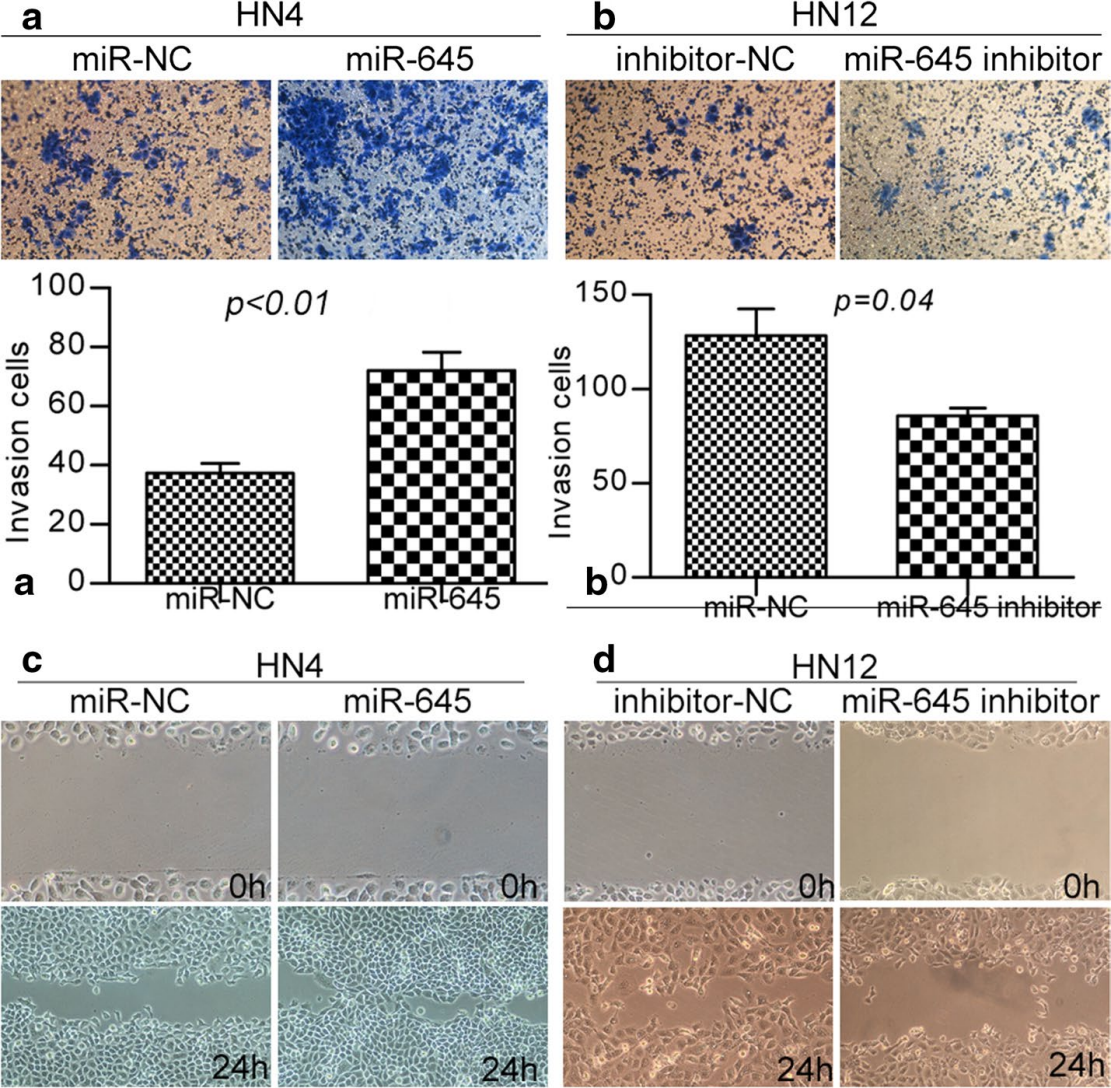
*miR*-*645* promotes cell invasion and migration. **a**, **b** Representative pictures of cells migrated through the filter, stained with crystal violet, and taken at the same magnification and absolute quantifications as cells that had invaded through the transwell. **c**, **d** Wound-healing assay showing that gain of *miR*-*645* promotes cell migration and loss of *miR*-*645* suppresses cell migration

In light of the preceding data, we aimed to determine more directly if *miR*-*645* plays a causal role in the aggressive traits of HNSCC cancer cells. We used this cell line to investigate how gain or loss of function of *miR*-*645* impacted cell migration and invasion, which are hallmarks of metastatic capacity. In the transwell assays shown in Fig. 3b, down regulation of *miR*-*645* in HN12 cells decreased invasive abilities 1.6-fold compared to the same cells expressing miR-NC. We further tested if endogenous expression of *miR*-*645* was required for cell invasion in HNSCC cancer cell line HN4. For this purpose, we upregulated *miR*-*645* and this treatment led to an approximately 1.8-fold augmentation in invasive properties (Fig. 3a). Furthermore, the pro-migration effects of *miR*-*645* were observed in wound-healing assays in HN4 and HN12 cells (Fig. 3c, d).

### Ectopic expression of *miR*-*645* promotes single cell clone proliferation

**Fig. 4:**
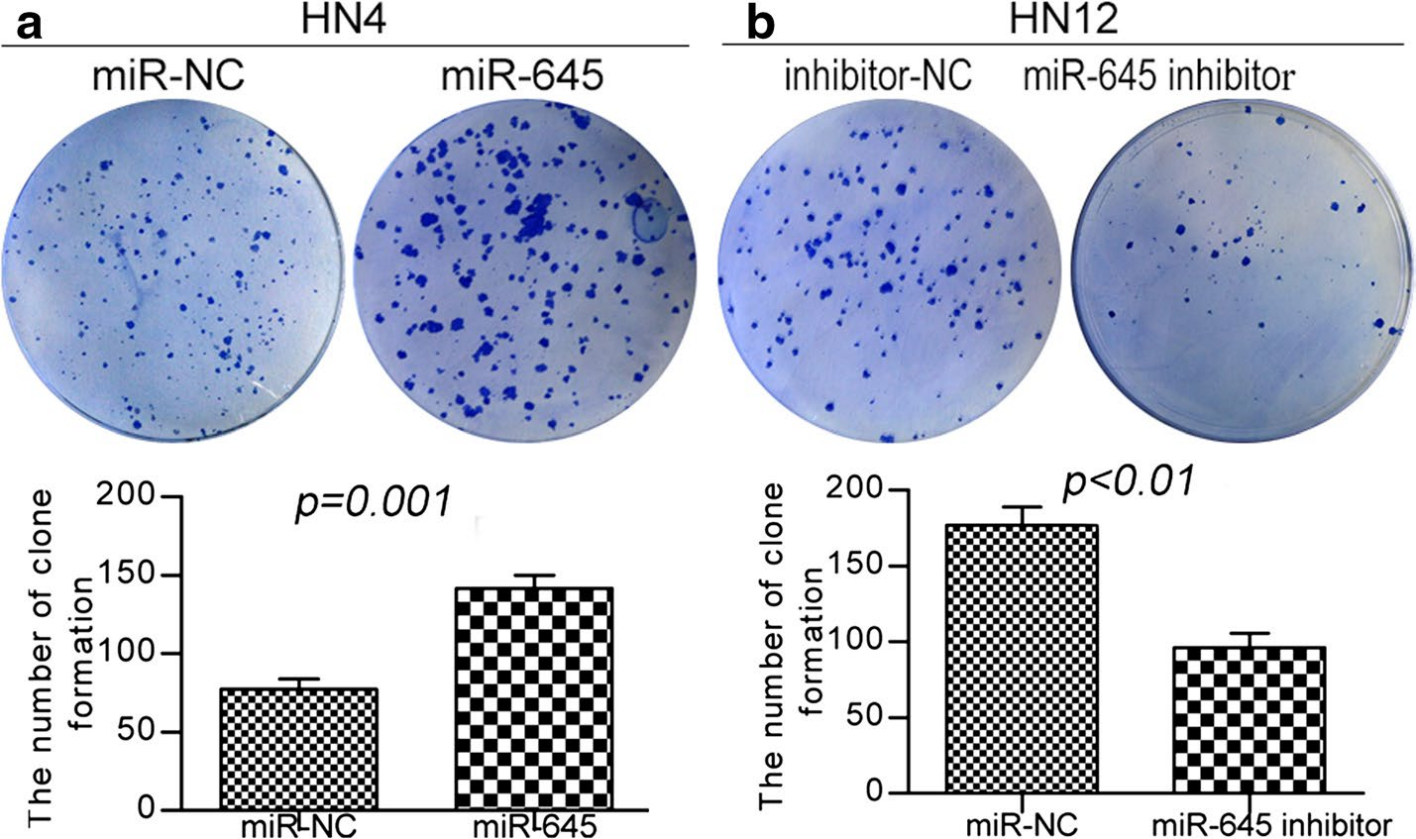
*miR*-*645* promotes single cell clone proliferation. **a**, **b** Representative pictures of single cell clone proliferation, stained with crystal violet, colony formation assay in the group of 1 × 105 cells for miR-NC transfected and miR-645-transfected cells

Single cell clone proliferation ability is considered to be hallmarks of metastatic capacity. We aimed to determine more directly if *miR*-*645* improve the single cell clone proliferation ability. We used this cell line to investigate how gain or loss of function of *miR*-*645* impacted the single cell clone proliferation ability. In the clony formation shown in Fig. 4a, up-regulation of *miR*-*645* in HN4 cells increased single cell clone proliferation ability 1.5-fold compared to the same cells expressing miR-NC. We further tested if endogenous expression of *miR*-*645* was required for cell invasion in HNSCC cancer cell line HN12. For this purpose, we down-regulated *miR*-*645* and this treatment led to an approximately 1.6-fold reduction in single cell clone proliferation properties (Fig. 4b).

## Discussion

Although a global reduction of miRNA abundance appears to be a general trait of human cancers, playing a causal role in the metastatic phenotype [11, 12, 31], several miRNAs are up-regulated in tumors [10], recently, miRNAs have been shown to be related to tumor metastasis [30], providing a new perspective on the metastatic process. Nonetheless, The role of miRNAs in HNSCC has been widely investigated. Here, we will focus on miRNA-645 promotes HNSCC cancer metastasis.

In this study, we described for the first time the *miR*-*645* is markly upregulated in metastatic HNSCC in large samples. We also found that *miR*-*645* is closely related to the cancer metastasis and *miR*-*645* “high expression” group displayed a significantly poorer disease-free survival rates. The *miR*-*645* promote the HNSCC cells proliferation, invasion, migration and the single cell clone proliferation ability. The properties is the malignant characters of oncogenes. We have proven that the down-regulation of *miR*-*645* is crucial in HNSCC metastasis and demonstrated that *miR*-*645* acts as a putative oncogene.

Recently, miRNAs have been proved to be related with tumor metastasis [32–34], providing a new perspective on the metastatic process. Nonetheless, the role of miRNAs in HNSCC metastasis is little known. This study first proves that *miR*-*645* is up-regulated in metastatic HNSCC. Aberrant patterns of miRNA expression are implicated in human diseases including HNSCC. *miR*-*645* plays a variety of important functions in physiology and pathology [22, 24]. Recent literature reports that the *miR*-*645* play important effort in the malignant progressing of ovarian cancer and adenocarcinoma of gastric esophagea [23, 25]. As miRNAs function mainly through the inhibition of multiple target genes and study report that miR-645 inhibits apoptosis by targeting tumor suppressor IFIT2 [25]. In our study, the inverse correlation between miR-645 and IFIT2 was confirmed by real-time PCR in the panel of 127 HNSCC tissues. We support that the IFIT2 may be the target gene of miR-645 **(**Fig. 2d). Meantime we support that the miRNAs play their efforts though the complicate gene net. This result may preliminary explain the function of *miR*-*645*, but the mechanism is still to be research.

## Conclusion

In conclusion, our results have proven that *miR*-*645* plays a causal role in the metastases of HNSCC. These findings have implications for understanding the mechanism of HNSCC, and *miR*-*645* may be a valuable maker and target for prevention or adjuvant therapy in HNSCC.
